# An Emergency Coordinated Control Strategy to Improve the Transient Stability of a Single-Ended Distribution Network with Flexible Interconnection Channel Blocking

**DOI:** 10.3390/s23208467

**Published:** 2023-10-14

**Authors:** Rui Ma, Haichang Sun, Liang Qin

**Affiliations:** 1 State Grid Jibei Electric Power Company, Beijing 100052, China; marui9015@foxmail.com; 2 School of Electrical Engineering and Automation, Wuhan University, Wuhan 430072, China; 2019302070148@whu.edu.cn

**Keywords:** AC/DC distribution network, blocking, coordinated control, improved PSO algorithm

## Abstract

Based on the scenario of high-penetration distributed photovoltaic connected to an AC/DC distribution network, this paper analyzes the dynamic characteristics of frequency and voltage in a distribution network after the blocking failure of the flexible interconnection channel. In order to enhance the transient stability of the system after the fault, this paper comprehensively considers the active regulation ability of photovoltaic units, and puts forward an emergency coordinated control strategy for a single-ended distribution network with flexible interconnection channel blocking. Firstly, the non-fault channel is overloaded for a short time, then the comprehensive influence of factors such as electrical distance, response time and adjustment cost on the frequency modulation effect of the system is quantitatively evaluated; according to the evaluation results, the photovoltaic and synchronous units are controlled by “control instead of tripping”, and finally, the high-frequency tripping is carried out, based on the principle of “photovoltaics first”. After the frequency control is completed, the reactive power optimization model of the system is established, and the improved tabu–particle swarm optimization algorithm is used to solve it, so as to optimize the voltage of the distribution network nodes. Finally, an equivalent simulation model is established to verify the coordinated control strategy.

## 1. Introduction

With the steady progress of the Chinese “carbon peaking and carbon neutrality goals” and the construction of a novel power system, the proportion of new energy in the power system is increasing; more and more distributed power sources are connected to the medium- and low-voltage distribution network nearby, and the traditional AC distribution network is gradually being upgraded to an AC/DC distribution network based on flexible interconnection technology [1]. However, the generation of high-penetration photovoltaic power and the high proportion of access to electronic equipment power will reduce the moment of inertia and damping of the distribution network, making the frequency adjustment and voltage support ability of the system weak [2].

When the flexible interconnection device in the AC/DC distribution network has an internal AC bus fault, a main circuit component fault, or a DC line disconnection fault, the system will block the flexible interconnection device. At this time, the frequency and voltage of the corresponding AC side will fluctuate violently. This sudden change in frequency and voltage may lead to the chain of disconnection of new energy units, which will have a greater impact on the stability of the system. Therefore, how to enhance the power control ability of the AC/DC distribution network with a high-penetration distributed power supply and improve the frequency and voltage stability of the novel power system is an urgent problem to be solved at the present.

Some scholars have carried out some research and exploration into the control technology of the blocking fault of flexible interconnection devices in an AC/DC distribution network with a high proportion of access to distributed new energy. Refs. [3,4,5] summarize the fault types of flexible interconnection devices in an AC/DC distribution network, but do not analyze the control strategy after the fault. Ref. [6] analyzes the transient process of an inverter-based distributed power supply when the distribution network fails, but it is only limited to the individual level of power supply. Refs. [7,8] analyze the frequency characteristics of the power system after the DC channel is disconnected and the problems it faces. Refs. [9,10] analyze the voltage variation characteristics after the DC channel disconnection, and put forward the calculation method for the transient overvoltage of an AC system after a fault.

Ref. [11] puts forward the primary frequency regulation control strategy of the system based on the analysis of the frequency response after the DC channel is disconnected, but it does not take into account the active regulation ability of new energy units. Jingzhe Tu, of the China Electric Power Research Institute, discussed the mutual restriction between distributed generation and a flexible interconnection system and the supporting role of synchronous units [12]. Shuyong Chen, of the China Electric Power Research Institute, put forward a control scheme for generator tipping, aiming at the goal of maintaining transient stability after system failure, pointing out that it is difficult to ensure stability or the cost is too high to just remove thermal power after a serious failure, so some distributed power sources should also be removed [13]. Yun Chen, of the North China Electric Power University, designed a scheme combining wind–photovoltaic–thermal energy with a high-frequency generator tripping to adapt to the fluctuation of wind speed and lighting [14]. Although the control strategies proposed by them partly involve the coordinated control of new energy units and conventional units, they only start from the point of view of generator tripping, and neither of them considers the power control of distributed power sources. Cutting off the new energy unit will cut off the reactive power source of the system accordingly, which will bring inconvenience to the voltage emergency control and the system will need to be connected to the grid again after the fault.

M Mohseni, of Curtin University in Australia, studied the voltage control strategy of a wind farm with a fault; the simulation showed that the strategy can make the wind farm meet the standards required by the Australian power grid [15], but did not consider establishing an optimization model to optimize the reactive power distribution of the system. H. Ahmad, of Tehran University in Iran, proposed that the influence of the power supply output fluctuation should be considered in the transient stability analysis of the power system with new energy units connected to the grid [16], but the voltage control strategy was not further studied.

It can be seen that at, present, there are relatively few research results on the coordination and cooperation between new energy units and conventional units in replacing the generator tripping with power control, and the research on the emergency coordinated control of node voltage by using new energy units is not deep enough.

In this paper, when the flexible interconnection device of AC/DC distribution network breaks down, an emergency coordinated control strategy of photovoltaic cluster considering the security and stability constraints of the distribution network is proposed. After the emergency coordinated control of frequency is completed following the principles of “control instead of tripping” and “photovoltaics first”, the optimal control of node reactive power is carried out, which realizes the optimal distribution and coordination of multiple active and reactive power sources in the distribution network and improves the stability of the system after the flexible interconnection device breaks down.

## 2. The Frequency and Voltage Characteristics of the Fault Area

**Figure 1:**
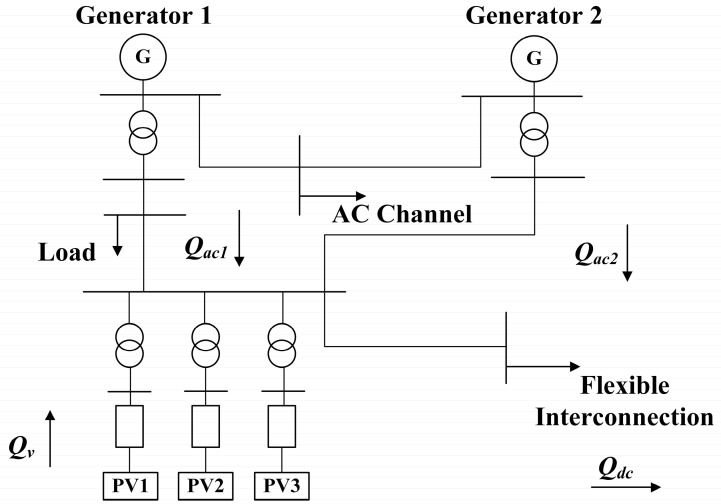
Typical AC/DC distribution network structure.

The typical structure of an AC/DC distribution network with high-penetration distributed photovoltaic access is shown in Figure 1.

Under normal working conditions, in order to effectively absorb new energy, reduce network loss, and realize mutual power assistance and dynamic capacity expansion between areas, all areas in the distribution network are interconnected on the low-voltage side, through flexible interconnection devices, to flexibly transmit power.

In the AC/DC distribution network, the breakdown of the interconnection devices may occur due to aging equipment, the design of secondary protection equipment without considering some special situations during the operation, and the influence between AC and DC systems, thus affecting the normal operation of the AC/DC distribution network.

During the fault period, the response characteristics of the different parts in the distribution network are also different. This section mainly studies the frequency and voltage-response characteristics of the single-ended distribution network with output power.

### 2.1. The Frequency Characteristics of the Fault Area

When the flexible interconnection device is disconnected, the active power transmission channel on the DC side is cut off. Excess power Δ*P* will appear in the fault area. The dynamic equation of the generator rotor is:

(1) $$J\frac{d(\Delta \omega /\omega_{0})}{dt}=P_{m}-P_{e}$$

where *P_m_* and *P_e_* are the mechanical power and electromagnetic power of the generator, respectively, *J* is the moment of inertia of the generator, Δ*ω* is the angular velocity deviation of the generator, and *ω*_0_ is the rated angular velocity of the generator.

In the dynamic transition process after the fault, each synchronous generator in the system distributes the disturbance quantity according to the moment of inertia. The change in the electromagnetic power of generator *i* in the system, according to the moment of inertia response disturbance, is as follows:

(2) $$\Delta P_{ei}(t)=-(J_{i}/\sum_{i=1}^{m}J_{i})\Delta P_{}$$

where *m* is the total number of synchronous units in the system, and *J_i_* is the moment of inertia of the *i*-th synchronous unit.

At this time, the electromagnetic power suddenly decreases, but the mechanical power cannot suddenly change, which causes the mechanical torque to be greater than the electromagnetic torque; this makes the angular speed of the generator increase, which in turn causes the frequency of the fault area to increase.

In order to explore the relationship between system frequency fluctuation and system inertia, the inertia time constant of the *i*-th generator with rated capacity of *S_gi_* is calculated as follows:

(3) $$H_{gi}=\frac{J_{i}\omega_{0}{}^{2}}{2S_{gi}}$$

However, photovoltaic units are connected to the grid through electronic power equipment, which does not involve the process of speed regulation and the inertial response of synchronous generators [17]. When the new energy with a rated capacity of *S_pv_* is connected, the equivalent inertia time constant of the distribution network is:

(4) $$H=\frac{\sum_{i=1}^{m}H_{gi}S_{g}}{S_{g}+S_{pv}}$$

It can be seen that the equivalent inertia time constant of the system will gradually decrease and the distribution network will gradually become a low-inertia system when the capacity of the connected new energy units increases.

When there is excess power in the fault area, when only the response of the synchronous unit and primary frequency regulation are considered, the system frequency change equation can be obtained from the dynamic equation of the generator rotor, as follows:

(5) $$\frac{2H}{f_{0}}\frac{df}{dt}=\Delta P_{g}-\Delta P-D(f-f_{0})$$

where Δ*P_g_* is the active power adjustment of the primary frequency modulation of the system, and *D* is the load damping coefficient.

By solving this equation, the equation of system frequency deviation can be obtained as [18]:

(6) $$\Delta f(t)=\frac{\Delta P}{K_{g}+D}+Ae^{\alpha t}(\sin\omega t+\phi )$$

where *K_g_* is the speed regulation gain of the unit, and *A* is the coefficient which is directly proportional to Δ*P* and inversely proportional to *H*.

By observing the structure of the above formula and finding the maximum value of frequency variation, it can be seen that, when the inertia of the system decreases and the same level of disturbance power is applied, the maximum value of frequency fluctuation will gradually increase [19].

Therefore, in the low-inertia system, when the excess power is large, serious high-frequency problems will appear in the fault area, and even lead to system frequency instability, which seriously threatens the security of the distribution network.

### 2.2. The Voltage Characteristics of the Fault Area

In the case of stable operation of the system, it is necessary to maintain the power exchange and balance among the photovoltaic, thermal power and DC interconnected systems. Figure 1 also shows the exchange of reactive power between the photovoltaic system and the DC interconnection system.

The reactive power balance of Bus 4 in the distribution network is as follows:

(7) $$Q_{dc}=Q_{ac1}+Q_{ac2}+Q_{v}$$

where *Q_dc_* is the reactive power output from the flexible interconnection device; *Q_v_* is the reactive power output from the photovoltaic system to the distribution network; and *Q_ac_*_1_ and *Q_ac_*_2_ are the reactive power output from the synchronous units.

After the flexible interconnection device breaks down, the *Q_dc_* drops to zero because the power transmission channel on the DC side is cut off.

At this time, a large amount of excess reactive power Δ*Q* appears on the AC side of the fault area. According to the power flow calculation formula, the voltage increment of AC bus in the fault area can be obtained as follows:

(8) $$\Delta U\approx \frac{PR+\Delta QX}{U_{N}}$$

Due to the large reactance parameter *X* in the distribution network, when there is large excess reactive power the overvoltage phenomenon will be more obvious; even the bus voltage will exceed the upper limit and the distributed photovoltaic will be off-grid because of the high voltage [20].

Therefore, the most fundamental way to eliminate the overvoltage problem caused by the blocking fault of the flexible interconnection device is to solve the problem of excess reactive power in the fault area.

## 3. The Basic Idea of Coordinated Control Strategy

As mentioned above, with the new energy with high penetration connected to the distribution network, the moment of inertia of the power system is reduced, due to the different characteristics of new energy from conventional energy, and the large power impact of the flexible interconnection device disconnection will bring great challenges to the stability of frequency and voltage of the distribution network. The traditional frequency regulation and voltage modulation measures can**no**t respond well to faults, which leads to system instability. It is necessary to re-study the frequency modulation and voltage regulation measures of the system.

The traditional frequency modulation and voltage regulation measures rely only on the removal of synchronous units, without considering the participation of photovoltaic units in the regulation. The removal of the synchronous unit will further reduce the inertia of the system, thus further affecting the original low-inertia system. However, photovoltaic power generation has good power regulation ability in essence, but it has not been fully tapped. It is connected to the grid through the inverter, and the active and reactive power control is flexible and responsive. Therefore, the advantages of photovoltaic units should be taken into account when controlling the system frequency and voltage.

Limited by the capacity of the photovoltaic unit, if it is used to adjust the system frequency and voltage at the same time, the adjustment effect may not meet the requirements of the control strategy. Because under normal working conditions photovoltaic units mainly output active power, and almost no reactive power, the premise of increasing the reactive power output is to reduce the active power output. At the same time, in order to ensure the absorption of new energy and reduce the waste of lighting, we should first reduce the active output of the unit and then adjust the reactive power, that is, control the frequency first and then control the voltage.

Therefore, in view of the frequency control problem, we should further fully tap the frequency modulation ability of photovoltaic energy. Based on the idea of “control instead of tripping”, through the cooperation between the photovoltaic unit’s participation in frequency modulation and the primary frequency regulation of conventional units, and, at the same time, taking advantage of the short-term overload ability of other power transmission channels to participate in system frequency modulation, we can formulate an emergency frequency control strategy that can simultaneously respond to the system frequency change and improve the problem of insufficient frequency modulation ability of the system.

After that, in order to solve the problem of excess reactive power in the fault area and restrain the rise in AC voltage, we should make full use of the advantages of active and reactive decoupling control and the rapid response of photovoltaic units to adjust the reactive power output of the photovoltaic units and to absorb excess reactive power in the fault area.

In order to optimize the reactive power output of the photovoltaic units, the reactive power optimization model of the distribution network should be established, and the model should be solved using the optimization algorithm to ensure that the voltage of each node is within a reasonable offset range.

**Figure 2:**
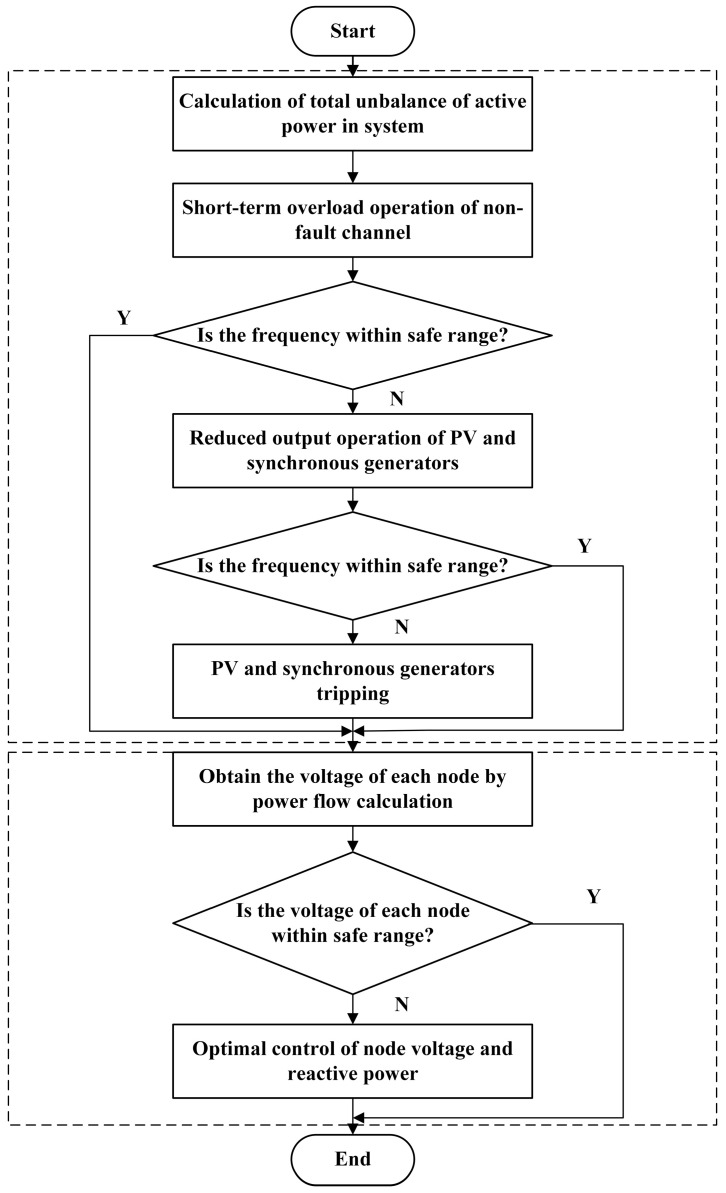
Flow chart of frequency–voltage coordinated control strategy.

To sum up, this paper puts forward a coordinated control strategy of frequency and voltage against the background of a blocking fault in the flexible interconnection channel. The specific process is shown in Figure 2.

## 4. Frequency Emergency Control Strategy

Because distribution lines generally have a certain multiple of overload capacity [21], when the flexible interconnection device is disconnected, the total active power unbalance of the system should be calculated first, and the non-fault AC and DC connection channels should be controlled for the short-term overload operation, so as not to reduce the output of each unit as much as possible.

However, the disconnection of flexible interconnection devices will bring about a great power impact, and it is often difficult to ensure the frequency stability of the system by simply relying on the overload operation of the channels.

If the output of new energy power generation can be reduced quickly after the fault, but it is not cut off, the increase in range of frequency and voltage can be controlled at the same time, and the complicated operation of photovoltaic reconnection can be avoided when recovering after the fault. Therefore, the effect of “control instead of tripping” is much better than that of simple tripping.

### 4.1. Factors Affecting Frequency Modulation of Output Reduction

In order to design a reasonable frequency modulation control strategy, we should first consider the influence of the regulating capacity of the photovoltaic and synchronous unit, the electrical distance to the flexible interconnection device and the response time and other factors on the frequency modulation effect of the system.

#### 4.1.1. Electrical Distance to Flexible Interconnection Device

Electrical distance can be used to measure the tightness of electrical connection between nodes in the system, and its definition is shown as follows:

(9) $$distance_{ij}=z_{ii}+z_{jj}-z_{ij}-z_{ji}$$

where *distance_ij_* is the electrical distance between node *i* and node *j*; *z_ii_* and *z_jj_* are the self-impedance of node *i* and node *j*, respectively; and *z_ij_* and *z_ji_* are mutual impedances between node *i* and node *j*.

The smaller the electrical distance between nodes, the closer the electrical connection of the system, and the more the branches put into operation in the system. On the contrary, the sparser the network, the more the branches which are out of service in the system.

In this paper, the equivalent model of a photovoltaic cluster near flexible interconnection devices in a regional distribution network is taken as an example to show the influence of electrical distance on system frequency modulation more intuitively. The basic parameters of the simulation model are shown in Table 1.

In the simulation model, the capacity of the superior power grid is 4000 kW, and it is assumed that three photovoltaic systems are merged into the same interconnection point through AC transmission lines with different lengths; the interconnection point is regarded as a faulty flexible interconnection device, so it can be considered that the ratio of the spatial distance between the three photovoltaic systems and the faulty flexible interconnection device is equal to the ratio of the electrical distance.

When other conditions of each photovoltaic unit are the same, but the electrical distance to the flexible interconnection device is different, the system load is set to decrease by 200 kW, step by step, at *t* = 5 s, and the changes in system frequency under various conditions are compared and analyzed.

**Figure 3:**
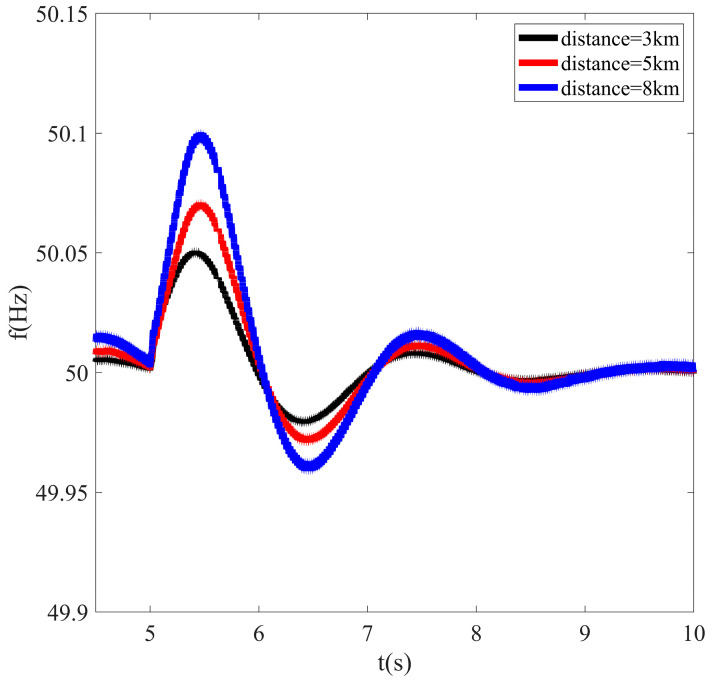
Influence of different electrical distances on system frequency modulation.

It can be seen from Figure 3 that the shorter the electrical distance between the photovoltaic unit with reduced output and the faulty flexible interconnection device, the better the support effect for system frequency modulation, and the more conducive it is to improving system frequency stability. Therefore, under the same other conditions, units with a shorter electrical distance should be set to undertake more power reduction.

#### 4.1.2. Regulation Capacity

The adjustable regulation capacity of unit *i* is defined as:

(10) $$P_{r}=P_{i}-P_{min-i}$$

where *P_i_* is the current output of active power of the *i*-th photovoltaic or synchronous unit and *P_min-i_* is the minimum output of active power of the *i*-th photovoltaic or synchronous unit.

In order to study the influence of regulation capacity on system frequency modulation, based on the above simulation model, different regulation capacities are set for three photovoltaic units, while other conditions of each photovoltaic unit are the same. The basic parameters of the simulation model are shown in Table 2.

The system load is still set to decrease by 200 kW, step by step, at *t* = 5 s, and the changes in system frequency under various conditions are compared and analyzed.

**Figure 4:**
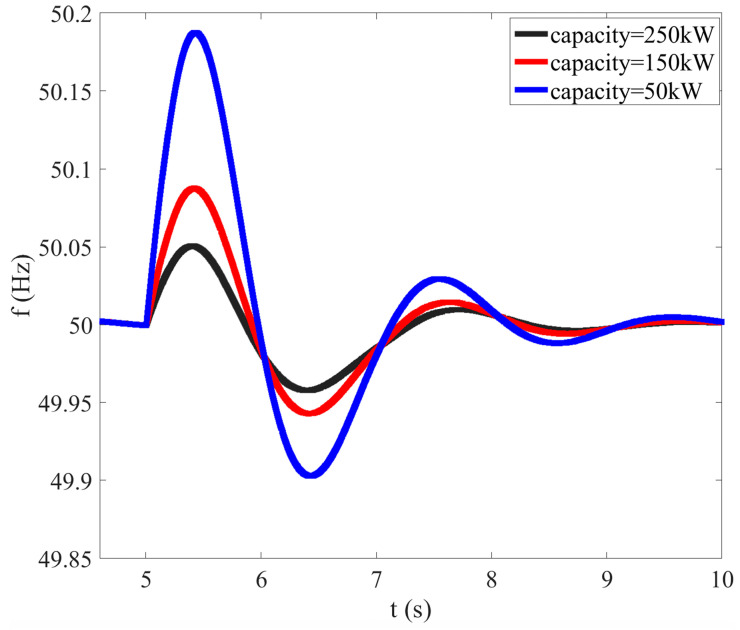
Influence of different regulation capacities on system frequency modulation.

As can be seen from Figure 4, when the surplus load is relatively large, the larger the adjustment capacity, the better the frequency modulation effect of the system.

Therefore, while the output reduction of each photovoltaic and synchronous unit is running, and when the total output reduction is certain, it should be distributed according to the adjustable capacity of each unit, so that the unit with a larger adjustable capacity can bear more output reduction, to ensure the frequency modulation effect of the system.

#### 4.1.3. Response Time and Adjustment Cost

The response time *τ_i_* of the *i*-th photovoltaic or synchronous unit reflects the speed of reducing the output of the unit.

In order to study the influence of response time on the system FM, based on the same simulation model, different response times are set for three photovoltaic units, while other conditions of each photovoltaic unit are the same. The basic parameters of the simulation model are shown in Table 3.

The system load is also set to decrease by 200 kW, step by step, at *t* = 5 s, and the changes in system frequency under various conditions are compared and analyzed.

**Figure 5:**
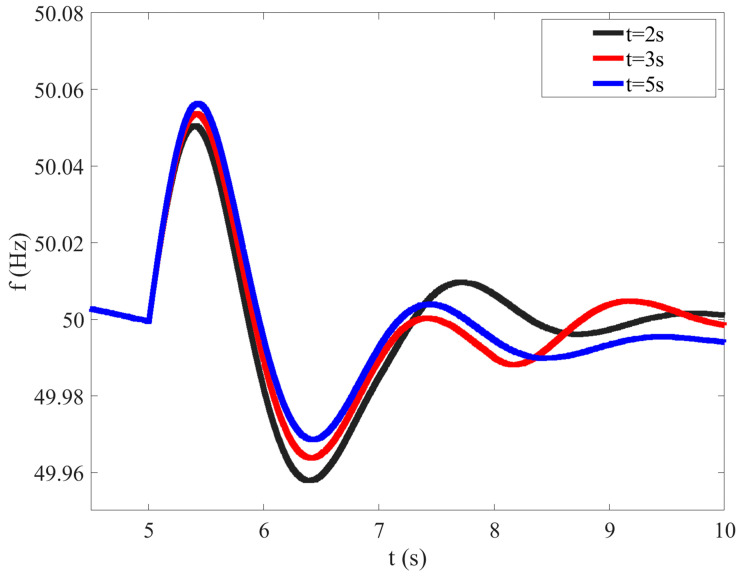
Influence of different response times on system frequency modulation.

As can be seen from Figure 5, the shorter the response time, the better the frequency modulation effect.

*Cost_i_* is the active power adjustment cost of the *i*-th photovoltaic or synchronous unit. The smaller the adjustment cost, the smaller the economic loss during frequency modulation.

Therefore, when the photovoltaic and synchronous units operate at reduced output, the units with shorter response time and lower adjustment cost should bear more reduced output power [22,23], so as to reduce the maximum frequency deviation, increase the frequency stability of the system and reduce the overall frequency modulation cost of the system.

### 4.2. Frequency Modulation Control Strategy of Output Reduction

#### 4.2.1. Comprehensive Distribution Index of Output Reduction

According to the above conclusions, when the photovoltaic and synchronous units are operated with reduced output, the adjustable capacity, the electrical distance to the faulty flexible interconnection device, the adjustment response speed, and the adjustment cost and other factors should be considered in the power distribution of each unit.

Therefore, in order to comprehensively evaluate the frequency modulation effect of unit output reduction and optimize the frequency modulation strategy of unit output reduction, this paper puts forward the comprehensive distribution index of photovoltaic and synchronous unit output reduction:

(11) $$C_{index-i}=\frac{P_{i}-P_{min-i}}{\tau_{i}\times Cost_{i}\times distance_{i}}$$

The greater the *C_index-i_* value, the better the frequency modulation effect of unit *i*. Therefore, after the failure of the flexible interconnection device, the unit should be allocated a greater share in the power reduction.

#### 4.2.2. Specific Steps of Output Reduction for Frequency Modulation

If the system frequency can**no**t reach the safety threshold, it is necessary to evaluate the photovoltaic and synchronous units according to the *C_index-i_* value, and then reduce the output.

At this point, set the output reduction power of the *i*-th photovoltaic or synchronous unit as:

(12) $$\Delta P_{i}=\frac{C_{index-i}}{\sum C_{index-i}}\times \Delta P$$

where Δ*P* is the total unbalance of system active power and Δ*P_i_* is the active power that should be reduced by the *i*-th photovoltaic or synchronous unit.

### 4.3. Frequency Modulation Strategy of Generator Tripping

When the output of the photovoltaic and synchronous units is reduced to the minimum, if it is still difficult to drop the system frequency to a safe and stable range, it is necessary to take shutdown measures for the photovoltaic and synchronous units.

#### 4.3.1. Comprehensive Index of Photovoltaic Unit Generator Tripping

When the photovoltaic unit is tripped, if other conditions are the same, the frequency modulation effect of the photovoltaic unit with a shorter electrical distance to the flexible interconnection device is better.

Therefore, this paper puts forward the comprehensive index of photovoltaic unit generator tripping to evaluate the comprehensive frequency modulation effect of photovoltaic unit generator tripping:

(13) $$\;C_{PV-i}=\frac{1}{distance_{i}}$$

where *C_PV-i_* is the comprehensive index of the photovoltaic generator tripping of the *i*-th photovoltaic unit and *distance_i_* is the electrical distance from the *i*-th photovoltaic unit to the faulty flexible interconnection device.

The greater the *C_PV-i_* value, the better the frequency modulation effect of photovoltaic unit *i*, so, after the fault, the unit should be given priority for being tripped.

#### 4.3.2. Comprehensive Index of Synchronous Unit Generator Tripping

The removal of the synchronous unit will cause instantaneous disturbance to the frequency of the system. When other conditions are constant, the smaller the electrical distance between the unit *i* and the faulty flexible interconnection device, *distance_i_*, the more intense the influence, and the worse the comprehensive frequency modulation effect of the corresponding generator tripping [24].

At the same time, according to the conclusion in Section 2, the removal of the synchronous generator will further reduce the equivalent inertia coefficient of the system, which will further affect the original low-inertia system and even cause system instability.

Therefore, the inertia time constant *H_i_* of the synchronous unit also has a great influence on the frequency modulation effect of the system. When *H_i_* is large, the removal of the synchronous unit will greatly reduce the equivalent inertia coefficient of the system and influence badly the stability of the system.

To sum up, this paper puts forward the comprehensive index of synchronous unit generator tripping to evaluate the comprehensive frequency modulation effect of a synchronous generator:

(14) $$C_{SM-i}=\frac{distance_{i}}{H_{i}}$$

where *C_SM-i_* is the comprehensive index of synchronous generator tripping of the *i*-th synchronous unit; *distance_i_* is the electrical distance from the *i*-th synchronous unit to the faulty flexible interconnection device; and *Hi* is the inertia time constant of the *i*-th synchronous unit.

The larger the *C_SM-i_* value, the better the frequency modulation effect of the synchronous unit *i*, so when this synchronous unit is tripped after the fault, it should be given priority.

#### 4.3.3. Specific Steps of Frequency Modulation of Generator Tripping

At this time, all photovoltaic units have been adjusted to the minimum active output, and they no longer have the adjustment capacity; the photovoltaic units generally show weak inertia or zero inertia. Therefore, the system shutdown process should start from photovoltaic units and follow the principle of “photovoltaics first”.

The photovoltaic units are tripped according to the comprehensive index *C_PV-i_* of photovoltaic generator tripping, in order from big to small.

When all photovoltaic units are tripped, if the system frequency is still not stable within the safety threshold it is necessary to cut off the synchronous units in the order of the comprehensive index of synchronous unit generator tripping *C_SM-i_*, until the system frequency returns to the safe range.

## 5. Voltage Emergency Control Strategy

After the emergency frequency control is completed, the reactive power distribution in the system is optimized by optimizing the reactive power output of reactive power sources such as the photovoltaic units and synchronous units, and the voltage is returned to the normal range.

### 5.1. Reactive Power Optimization Model of Distribution Network

The voltage of each node of the system can be obtained by power flow calculation for the AC/DC distribution network. In order to eliminate the problem of voltage exceeding limits and to optimize the voltage distribution of the distribution network nodes, this paper establishes the reactive power optimization model of the distribution network:

The objective function of the optimization model is:

(15) $$\min U_{error}=\sum_{t=1}^{T}{\left(U_{i}-1\right)}^{2}$$

The objective function of the optimization model is to minimize the deviation of each node’s unit voltage, that is, each node’s voltage should be as close as possible to the node’s voltage reference value, so as to reduce the risk of voltage exceeding the limit and to increase the system’s voltage stability.

Constraints:

i. Reactive power output constraint of each node;

(16) $$Q_{i}^{\min}\leq Q_{i}\leq Q_{i}^{\max}$$

where $Q_{i}^{\min}$ is the minimum reactive power output from the node; the photovoltaic node inverter can absorb reactive power due to the complete decoupling of active and reactive power control, so the $Q_{i}^{\min}$ can be negative; $Q_{i}^{\max}$ is the maximum reactive power that each node can output under the current weather conditions. This constraint condition ensures that the reactive power output of each node meets the reactive power limit of the node.

ii. Power flow equality constraints of each node:

(17) $$\{\begin{array}{c} \begin{array}{l} P_{gi}-P_{di}-U_{i}\sum_{j\in i}U_{j}\left(G_{ij}\cos\theta_{ij}+B_{ij}\sin\theta_{ij}\right)=0 \end{array}\\ Q_{gi}-Q_{di}-U_{i}\sum_{j\in i}U_{j}\left(G_{ij}\sin\theta_{ij}-B_{ij}\cos\theta_{ij}\right)=0 \end{array}$$

where *P_gi_*, *P_di_*, *Q_gi_*, *Q_di_* and *U_i_*, respectively, represent the active output, active load, reactive input, reactive load and node voltage of node *i*; *G_ij_*, *B_ij_* and *θ_i_*, respectively, represent the conductance, susceptance and phase-angle difference of branch *i-j*.

iii. Voltage constraint of each node:

(18) $$\;U_{imin}\leq U_{i}\leq U_{imax}$$

where *U_imin_* and *U_imax_* are the upper- and lower-voltage limits of bus *i*.

### 5.2. Solving Algorithm

In this paper, the improved tabu–particle swarm optimization algorithm is used to solve the optimization model and find the optimal solution of the model.

In the conventional particle swarm optimization algorithm, after the initialization of particle attributes, the algorithm updates the velocity and position attributes through Equations (19) and (20), respectively, in the iterative process [25] in order to become closer to the global optimal solution.

The d-dimensional velocity updating formula of particle *i* is:

(19) $$\begin{array}{l} \;v_{id}^{k}=\omega v_{id}^{k-1}+c_{1}r_{1}\left(pbest_{id}-x_{id}^{k-1}\right)\\ +c_{2}r_{2}\left(gbest_{d}-x_{id}^{k-1}\right) \end{array}$$

The formula for updating the dimension position of particle *i* is:

(20) $$\;x_{id}^{k}=x_{id}^{k-1}+v_{id}^{k-1}$$

where *v_kid_* is the d-dimensional flight velocity vector of particle *i* after the *k*-th iteration; *x_kid_* is the d-dimensional position vector of particle *i* after the *k*-th iteration; and *c_1_* and *c_2_* are learning factors, that is, acceleration constants. The former represents self-experience and the latter represents group experience, which are used to adjust the maximum step length of learning. *r_1_* and *r_2_* are random probability values, and random values between [0, 1] are taken to increase the randomness of the algorithm search; *w* is the inertia weight, and the value range is [0, 1], which is used to adjust the search range of the solution space.

The disadvantage of the conventional particle swarm optimization algorithm is that its convergence speed is obviously slow in the late iteration process, and it is easy to fall into the local optimal solution.

In order to improve the local optimization ability of the algorithm in the later iteration process and achieve the global optimal solution more easily, this paper proposes a linear decreasing inertia weight strategy to improve the particle swarm optimization [26].

(21) $$\;w=w_{max}-\frac{(w_{max}-w_{min})\times n}{G}$$

where *w_max_* and *w_min_* are the maximum and minimum inertia weights, respectively, *n* is the current iteration number, and *G* is the total iteration number of the PSO algorithm.

The inertia weight *w* decreases with the increase of iteration times, and in the early stage of iteration the inertia weight value is large, which ensures its original global search ability; in the later stage of the iterative process, the inertia weight value is small, which enhances the local optimization ability and avoids falling into the local optimal solution.

However, when the nonlinear optimization model is complex, it is still difficult to avoid premature convergence and dimension disaster simply by relying on the adaptive optimization ability of particle swarm optimization [27].

Therefore, this paper considers the advantages of the tabu search algorithm to effectively avoid falling into the local optimal solution [28], and combines the advantages of particle swarm optimization (PSO) in the early stage of convergence, and putting forward an improved tabu–PSO algorithm, the specific steps of which are as follows [29]:

(a) Firstly, the improved particle swarm optimization algorithm is used to solve the initial optimization iteration, and after the initial convergence to the current optimal value and repeated *PSO_min* (local small search times, which can be taken as 50 in this model), the current optimal solution is searched carefully in a small neighborhood; after converging to the current optimal value, repeat *PSO_max* (the maximum repetition value can be taken as 100 in this model) times or reach the maximum iteration number of particle swarm optimization algorithm, then store the current local optimal solution in the tabu list;
(b) Performing a large neighborhood search on the current local optimal solution to obtain a local suboptimal solution as the initial value of the next PSO algorithm optimization;
(c) Repeating step (a) and (b) for the global maximum number of iterations;
(d) Searching the tabu list to find out the global optimal value from a plurality of local optimal solutions.

**Figure 6:**
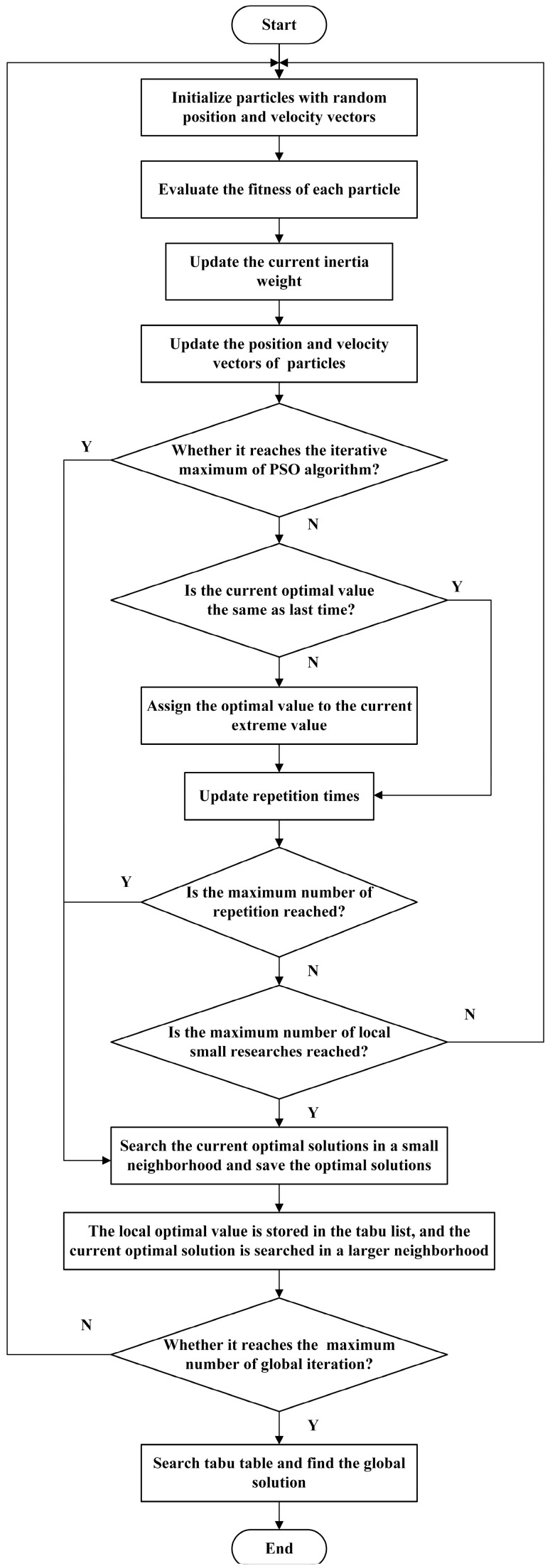
Flow chart of improved tabu–particle swarm optimization algorithm.

The algorithm flow chart is shown in Figure 6.

## 6. Simulations

### 6.1. Model Parameters

**Figure 7:**
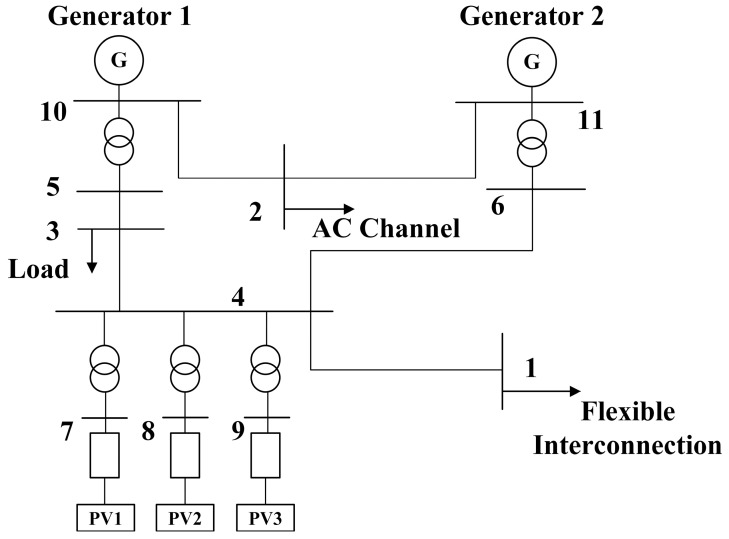
Topological structure of the equivalent simulation model.

In order to verify the effectiveness of the emergency coordinated control strategy of system frequency and voltage when the flexible interconnection device is disconnected in the scenario of high-penetration photovoltaic access to an AC/DC distribution network, an equivalent model, as shown in Figure 7, is established by taking a certain regional AC/DC distribution network as an example. There are 11 nodes in the equivalent model and the specific parameters are shown in Table 4, Table 5, Table 6, Table 7, Table 8, Table 9 and Table 10.

- Parameters of photovoltaic and synchronous generator
- Bus data
- AC line data
- Transformer data
- Data of power generation nodes
- Data of load nodes
- Overload capacity of AC and DC delivery channels

### 6.2. Simulation Results

When the flexible interconnection channel is blocking, unbalanced active power is generated in the system. Therefore, according to the coordinated control strategy proposed in this paper, the output reduction and tripping of each photovoltaic and synchronous unit are shown in Table 11.

When the flexible interconnection channel is broken, the output of each photovoltaic and synchronous unit is reduced, according to Table 11, and the voltage of each node of the system is shown in Table 12.

Taking the output of reactive power of each PQ node as the control object, the voltage of each node is optimized according to the optimization model established above, and the output of reactive power of each PQ node after optimization is shown in Table 13.

The optimized node voltages are shown in Table 14.

**Figure 8:**
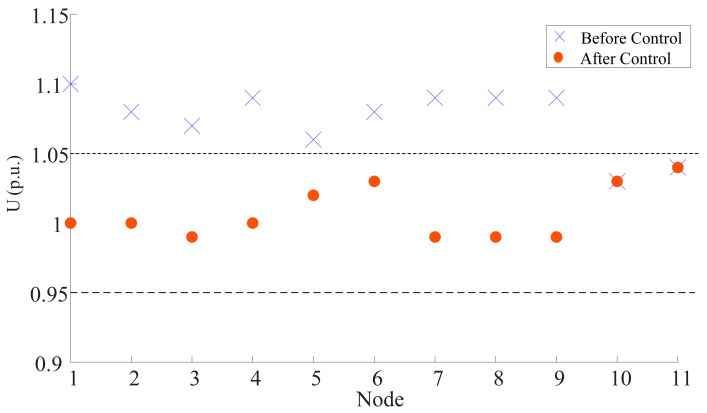
Voltage comparison of each node before and after reactive power optimization.

The comparison diagram before and after voltage optimization of each node is shown in Figure 8.

As can be seen from Figure 8, after the reactive power optimization according to the cooperative control strategy proposed in this paper, the voltage of each node of the distribution network has returned to the allowable deviation range, and the problem of the voltage of the distribution network exceeding the limit after the flexible interconnection device is disconnected is effectively solved.

## 7. Conclusions

For the AC/DC distribution network with high-penetration distributed photovoltaic access, when the flexible interconnection device breaks down, the main problems faced by the photovoltaic access area are as follows: firstly, the energy impact caused by the disconnection of the DC interconnection channel makes the power grid generate a large amount of excess power, and the frequency will fluctuate violently or even become unstable. It is necessary to deliver excess power in time and reduce the output of the unit, and even shut down the units. Secondly, due to the excess reactive power, the voltage will gradually rise, even beyond the limit. It is necessary to give full play to the advantages of the photovoltaic units and to absorb excess reactive power.

When reducing the output of the photovoltaic and synchronous units for frequency modulation, the unit with the shorter electrical distance from the flexible interconnection device and a shorter response time and lower adjustment cost has a better frequency modulation effect, so such units should be set to undertake more output reduction of power. When frequency modulation is carried out by generator tripping, the principle of “photovoltaics first” should be followed, and photovoltaic units should be shut down first.

The simulation results show that the emergency coordinated control strategy based on “replacing switching with control” and “photovoltaics first” proposed in this paper can effectively maintain the frequency and voltage stability of a distribution network.

## Figures and Tables

**Table 1:** The parameters of the simulation model for studying the influence of electrical distance.

PV Unit	Regulation Capacity	Response Time	Spatial Distance
PV 1	250 kW	2 s	3 km
PV 2	250 kW	2 s	5 km
PV 3	250 kW	2 s	8 km

**Table 2:** The parameters of the simulation model for studying the influence of regulation capacity.

PV Unit	Regulation Capacity	Response Time	Spatial Distance
PV 1	250 kW	2 s	3 km
PV 2	150 kW	2 s	3 km
PV 3	50 kW	2 s	3 km

**Table 3:** The parameters of the simulation model for studying the influence of response time.

PV Unit	Regulation Capacity	Response Time	Spatial Distance
PV 1	250 kW	2 s	3 km
PV 2	250 kW	3 s	3 km
PV 3	250 kW	5 s	3 km

**Table 4:** Basic parameters of photovoltaic and synchronous generator.

Units	Minimum Active Power Output (p.u.)	Response Time of Active Power Regulation (s)	Cost of Active Power Regulation (1/p.u.)	Inertial Time Constant (s)	Electrical Distance to Flexible Interconnection Device (p.u.)
PV 1	0.12	3	0.2	-	0.0634
PV 2	0.1	3	0.24	-	0.0651
PV 3	0.03	2	0.16	-	0.0484
Generator 1	0.27	15	1	13.9	0.0092
Generator 2	0.18	20	0.85	11.9	0.0292

**Table 5:** Bus data.

Bus Number	Reference Voltage(kV)
1	0.4
2	10
3	0.4
4	0.4
5	0.4
6	0.4
7	0.22
8	0.22
9	0.22
10	10
11	10

**Table 6:** AC line data.

I-Side Bus	J-Side Bus	Resistance (p.u.)	Reactance (p.u.)	1/2 of Susceptance (p.u.)
6	1	0.01	0.085	0.088
1	4	0.032	0.161	0.153
4	3	0.0085	0.072	0.0745
3	5	0.0119	0.1008	0.1045
10	2	0.039	0.17	0.179
2	11	0.017	0.092	0.079

**Table 7:** Transformer data.

I-Side Bus	J-Side Bus	Reactance (p.u.)
11	6	0.0576
10	5	0.0586
7	4	0.0625
8	4	0.0625
9	4	0.0625

**Table 8:** Data of power generation nodes.

Bus Number	Bus Type	Active Power(p.u.)	Reactive Power (p.u.)	Voltage (p.u.)	Voltage Phase Angle
11	Swing	-	-	1.04	0
7	PQ	0.96	0	-	-
8	PQ	0.8	0	-	-
9	PQ	0.24	0	-	-
10	PV	1.2	-	1.025	-

**Table 9:** Data of load nodes.

Bus Number	Bus Type	Active Power (p.u.)	Reactive Power (p.u.)
1	PQ	2.5	0
2	PQ	1.5	0.3
3	PQ	1	0.35

**Table 10:** Overload capacity of AC and DC delivery channels.

Delivery Channels	Bus Number	Rated Power	Overload Ratio
Flexible interconnection channel	1	250 kW	1.1
AC channel	2	100 kW	1.1

**Table 11:** Output of each unit under flexible interconnection blocking fault.

Operation Condition	PV1	PV2	PV3	Generator 1	Generator 2	AC Transmission
Normal condition	96 kW	80 kW	24 kW	180 kW	120 kW	250 kW
Blocking fault	12 kW	12.2 kW	3 kW	129.6 kW	110.7 kW	267.5 kW

**Table 12:** Voltage of each node under flexible interconnection blocking fault.

Bus Number	Voltage (p.u.)
1	1.10
2	1.08
3	1.07
4	1.09
5	1.06
6	1.08
7	1.09
8	1.09
9	1.09
10	1.03
11	1.04

**Table 13:** Reactive power optimization results of each node.

Bus Number	Reactive Power Output (kVar)
1	0
2	0
7	−7.11
8	−7.11
9	−7.16

**Table 14:** Voltage of each node after reactive power optimization.

Bus Number	Voltage (p.u.)
1	1.00
2	1.00
3	0.99
4	1.00
5	1.02
6	1.03
7	0.99
8	0.99
9	0.99
10	1.03
11	1.04

## Data Availability

Not applicable.
